# Reducing the Fermentability of Wheat with a Starch Binding Agent Reduces Some of the Negative Effects of Heat Stress in Sheep

**DOI:** 10.3390/ani12111396

**Published:** 2022-05-28

**Authors:** Pragna Prathap, Surinder S. Chauhan, Brian J. Leury, Jeremy J. Cottrell, Aleena Joy, Minghao Zhang, Frank R. Dunshea

**Affiliations:** 1 Faculty of Veterinary and Agricultural Sciences, The University of Melbourne, Parkville, VIC 3010, Australia; pragna.prathap@student.unimelb.edu.au (P.P.); ss.chauhan@unimelb.edu.au (S.S.C.); brianjl@unimelb.edu.au (B.J.L.); jcottrell@unimelb.edu.au (J.J.C.); aleenajoyj@student.unimelb.edu.au (A.J.); minghao@student.unimelb.edu.au (M.Z.); 2 Faculty of Biological Sciences, The University of Leeds, Leeds LS2 9JT, UK

**Keywords:** Bioprotect, heat stress, nutritional interventions, physiological responses, thermotolerance

## Abstract

**Simple Summary:**

Heat stress is one of the major problems affecting the thermotolerance of livestock in Australia and in many parts of the world. Dietary interventions are considered to be one of the effective strategies to beat the heat. Hence, this study evaluated the benefits of reducing the fermentability of wheat grains (Bioprotect^TM^) in the diet of Merino lambs. These results indicated that Bioprotect supplementation had a positive effect on the respiratory rate and heart rate of heat-stressed lambs.

**Abstract:**

The objective of this study was to investigate the effects of reducing the fermentability of grains on thermoregulatory responses in heat stressed (HS) lambs. To achieve this, wheat grain treated with a commercial starch binding agent, Bioprotect, is compared to maize, which has already demonstrated effects in ameliorating heat stress-induced thermoregulation responses and untreated wheat grains. An initial in vitro experiment was conducted to examine cumulative gas production from the fermentation of wheat grain with different dosages of the commercial starch binding agent, Bioprotect. Based on the in vitro results, an in vivo lamb experiment was conducted using 24 Merino lambs (1 year old; 42.6 ± 3.6 kg BW). The lambs were offered one of three dietary treatments: a wheat-based diet (WD), a Bioprotect treated wheat-based diet (BD), and a maize-based diet (MD). Three successive 1-week experimental periods were conducted with lambs from all dietary groups (P1, P2, and P3). During P1, lambs were exposed to a TN environment and fed a 1.7× Maintenance feed intake (MF) level; in P2, lambs were kept in a HS environment and fed a 1.7× MF level; and in P3, animals were kept in a HS environment and fed a 2× MF level. The in vitro experiment revealed a reduction in cumulative gas production (*p* < 0.05) from the Bioprotect treated wheat compared to untreated wheat samples. In the in vivo component of the study, the replacement of wheat with maize or 2% Bioprotect-treated wheat reduced the respiration rate (*p* < 0.001) and heart rate (*p* ≤ 0.01) of lambs during HS. There was a reduction in the concentration of blood gas variables such as a base excess of blood (BE(b)) and extracellular fluid (BE(ecf)), bicarbonate (CHCO_3_^−^), the partial pressure of carbon dioxide (pCO_2_), the total concentration of carbon dioxide (ctCO_2_), and sodium (Na^+^) (*p* ≤ 0.001 for all) during the periods of HS compared to the thermoneutral conditions. Moreover, BD- and MD-fed lambs had a higher blood potassium concentration (K^+^) than the WD-fed lambs (*p* = 0.008). The results of the present study suggest that Bioprotect can be a viable feed treatment strategy for treating rapidly fermentable grains such as wheat to alleviate the effects of HS. Further, Bioprotect-treated wheat could be an option to replace maize in concentrate rations in jurisdictions where maize is cost-prohibitive or unavailable.

## 1. Introduction

Continuous selection for high-yielding wool and meat traits has made the sheep population more susceptible to summer heat stress (HS) due to associated feed and metabolic energy requirements [1,2]. Many studies have reported a HS-associated elevation in the thermoregulatory responses [3,4,5] and a decline in the production parameters of sheep [6]. Often, there are reports of impaired reproduction variables such as pregnancy rate, embryo survival, and lambing rates [7,8]; therefore, researchers and farmers have increased attention into the use of feed or feed additives that can mitigate the seasonal effects of heat while using livestock with a high genetic gain [9,10]. In addition, the deterioration of pasture quality during the summer requires farmers to supplement their animals with grains and silages to maintain growth [11]. Studies have shown that concentrate supplementation is an effective means to regulate HS [12,13]. However, the presence of highly fermentable grain such as wheat in the concentrate diet can offset the benefit of increasing the concentrate content of the feed [14]. Wheat is the commonly used grain in the feed of Australian livestock and many parts of the world. Previous studies have identified that feeding slowly fermentable grain such as maize can reduce the metabolic heat load during thermal stress conditions in sheep [15,16]. In countries such as Australia, where maize production is lower and the grain is often too costly to use as an animal feed, there is a need for alternative options for slowly fermentable grain. In this context, finding alternatives to maize could be beneficial to lamb production. In vitro studies have shown that a starch binding agent (Bioprotect™, RealisticAgri, Ironbridge, UK) can reduce the rate of fermentation of wheat [17]. Bioprotect is a commercially available starch and protein binding agent. Various chemical components present in Bioprotect bind the starch and protein components of feed, thereby reducing rumen fermentation. The complexes with Bioprotect are stable at rumen pH but dissociate in the acidic environment of the abomasum, thus rendering the starch component in the feed matrix available for post-ruminal enzymatic digestion [18,19]. In addition, Bioprotect can reduce the metabolic heat production to promote HS alleviation. Furthermore, replacing wheat with wheat treated with the starch binding agent increased milk yield and milk fat % in dairy cows in summer [20]. Therefore, the objective of this study was to evaluate the amelioration potential of the starch binding agent (Bioprotect™)-treated wheat grain and maize grain over wheat grain and to observe the impact of HS on the thermoregulatory responses of sheep.

## 2. Materials and Methods

### 2.1. In Vitro Gas Production

An in vitro gas production experiment was performed to compare the total gas production from different dosages of Bioprotect-treated wheat (RF gas production system, ANKOM Technology, Macedon, NY, USA). Four inclusion rates of Bioprotect were evaluated: 0% Bioprotect, 1% (10 L/tonne), 2% (20 L/tonne), and 4% (40 L/tonne). Different dosages of Bioprotect were prepared by thoroughly mixing the Bioprotect with 100 g of ground wheat (particle size ≤ 1 mm). To ensure the proper mixing of Bioprotect, a single drop at a time was placed using a 1 mL syringe onto the grain, and it was mixed thoroughly with the aid of fingers in an aluminium foil tray. After the mixing, the samples were rested for 48 h. After the resting period, 1 g of sample was weighed into the 250 mL fermentation bottles. Each treatment had 6 replications in one fermentation run. Altogether, four fermentation runs were carried out using the rumen fluid sourced from beef cattle from an abattoir. Rumen fluid was transported from the abattoir to the laboratory in a glass bottle with a one-way valve and kept in a portable incubator maintained at 39 °C. Immediately after reaching the laboratory, rumen fluid was strained using a 4-layer cheesecloth and flushed with CO_2_ to maintain an anaerobic atmosphere. Then, the rumen fluid was buffered using Kansas State buffer, pH 6.8 at a 3:1 ratio [21,22,23]. Then, 100 mL of buffered rumen fluid was dispensed into the fermentation bottles containing feed samples. Additionally, each fermentation run had 6 blank bottles (only buffered rumen fluid) without any feed samples to correct for background gas production [24,25]. All the procedures involving rumen fluid manipulation were performed under anaerobic conditions with the flushing of CO_2_ gas. Later, all the bottles were randomly stacked in the water baths at 3 °C and left for 24 h. The wireless ANKOM RF gas production system can measure and transmit internal gas pressure every 5 min to the associated laptops. At the end of the 42 h run, cumulative gas production in kPa was converted to mL/g DM.

### 2.2. Animals

All the experimental procedures involving animals were performed in accordance with the guidelines from the Animal Ethics Committee (Ethics ID:1914950.1) of the Faculty of Veterinary and Agricultural Sciences, The University of Melbourne. Twenty-four 1-year-old Merino lambs (42.6 ± 3.6 kg BW) were sourced from the university’s Dookie College sheep flock based on their body weight and age. Following initial adaptation to indoor housing for 1 week and individual penning for 15 days, all the sheep were placed in individual metabolic cages for a further 3 days acclimatization before the experimental period commenced. During the 15 days individual pen adaption period, animals were introduced to the grains, gradually increasing the grain intake by 50 g/day until the required rate. Using a randomized control design, animals were allocated to 3 different diets, the control diet (WD; n = 8) was composed of 50% rolled wheat grain and 50% roughage (25% Oaten chaff and 25% Lucerne chaff), and two intervention diets, one consisting of 50% rolled maize grain and 50% roughage (25% Oaten chaff and 25% Lucerne chaff; MD; n = 8) and the other one composed of rolled wheat grain treated with 2% Bioprotect and 50% roughage (25% Oaten chaff and 25% Lucerne chaff; BD; n = 8). The ingredient and chemical composition of the experimental diets are given Table 1. Wheat grain was treated with 2% Bioprotect with the help of a cement mixer by continual spraying a small quantity using a water sprayer while mixing the grain. The mixture was rested for at least 3 days in plastic bags. The results of an in vitro rumen gas production experiment determined that 2% Bioprotect was an effective dosage. The daily feed requirement was calculated using the formula as cited by [26]. Briefly, feed offered (kg DM/day) = W^0.75^ × 450/1000/ME, in which W^0.75^ is the metabolic body weight of the animal, 450 is the maintenance energy requirement for sheep (kJ/kg W^0.75^), and ME is the metabolizable energy content of the diet. The daily feed ration was split into two; morning ration at 09:00 h, and afternoon ration at 13:00 h.

The animals underwent 3 different experimental periods, period 1 (P1) consisted of 7 days of thermoneutral conditions (18–21 °C and 40–50% relative humidity; RH) and feed 1.7× Maintenance feeding level; period 2 (P2), consisted of 7 days of cyclic HS (HS; 28–38 °C and 30–50% RH) and 1.7× Maintenance feeding level; and Period 3 (P3), consisted of 7 days of cyclic HS as in P2 with 2× Maintenance feeding level. In the HS room, the temperature was increased to 38–40 °C between 08:00 and 17:00 h daily and maintained at 28 °C for the remainder of the day. Drinking water was offered ad libitum.

### 2.3. Variables

#### 2.3.1. Body Weight, Water, and Feed Intake

Bodyweight was measured using a walk-over weighing balance in the morning before commencing the experiment and after the end of each period. Feed intake and water disappearance were measured every day at 08:30 h by measuring the leftover from the previous day.

#### 2.3.2. Blood Gas and Physiological Variables

Physiological responses were recorded thrice daily (09:00, 12:00, and 16:00 h) using standard methods. Respiration rate (RR) was measured by carefully observing the flank movements for 20 s and then converted to breaths/minute. Heart rate (HR) was determined using a stethoscope (3M Littmann Master Classic; 3M Health Care, St. Paul, MN, USA) by counting the number of beats in 20 s and then converted to beats per minute. Rectal temperature (RT) was measured with the help of a digital thermometer (Welcare Digital Thermometer Ultimate; Hangzhou Hua’an Medical and Health Instruments Co., Ltd., Zhejiang, China). Left and right flank temperature (LFT and RFT) were measured by exposing the skin after separating wool.

Blood samples were collected at the end of each period (13:00 h) from the external jugular vein using 19-gauge sterile needles and plastic syringes. The blood gas and acid-base analysis were performed using self-calibrated portable blood analyzer (EPOC BGEM Blood Analysis System; Alere, Inc. Analytical variables determined were blood pH, base excess of blood (BE(b)), base excess of extracellular fluid (BE(ecf)), the calculated values of bicarbonate (CHCO_3_^−^), partial pressure of CO_2_ (pCO_2_), partial pressure of O_2_ (pO_2_), Na^+^, K^+^, and total CO_2_ (ctCO_2_).

### 2.4. Temperature-Humidity Index and Measurement

**Figure 1:**
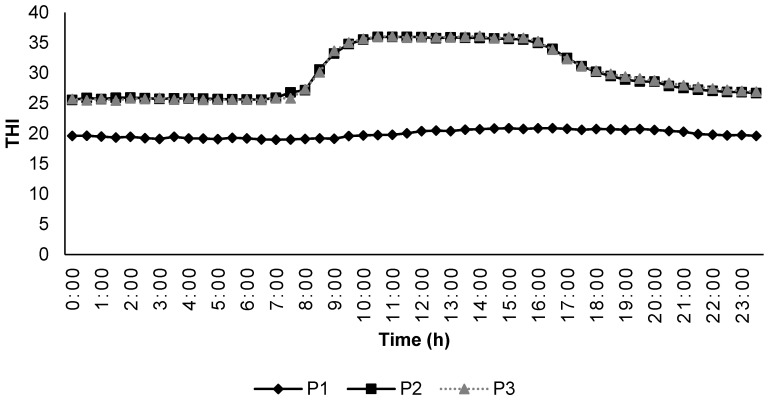
Temperature humidity index inside the climatic chambers during the experimental periods.

During the experiment, the temperature and humidity inside the climatic chambers were measured with the help of a USB temperature–humidity data logger (Instrument Choice, Dry Creek, Adelaide, Australia) at 30-min intervals. The temperature–humidity index was calculated by the formula described by [27] using the equation THI = [T − (0.31 − 0.0031 × RH) × (T − 14.4)], where T is the ambient temperature (°C) and RH is the relative humidity (%). Any THI values below 22.2 were classified as the absence of HS; THI between 22.2 to <23.3 as moderate HS; THI between 23.3 to <25.6 as severe HS; and 25.6 and more as extreme severe HS. The changes in THI during the different experimental periods are included in Figure 1.

### 2.5. Statistical Analysis

All statistical analyses were performed using ANOVA or REML variance component analysis procedures in Genstat (GenStat 16th edition, VSN International Ltd., Hemel Hempstead, UK). For the in vitro experiment, the REML fixed model effects were Time (0, 4, 8, 12, 16, 20, and 24 h), Bioprotect (0 vs. pooled 10, 20, and 40 g/kg) and within Bioprotect (10 vs. 20 vs. 40 g/kg) and random effects were flask number and replication. The data were also analysed by ANOVA suitable for a dose-response study with the main effects of time and linear and quadratic effects of dose of Bioprotect and their interactions with flask number and replication as blocking factors. In the animal study for the daily measurements (physiological variables, water disappearance, and feed intake), the REML fixed model effects were diet, period, and day and random effects were lamb ID and replication. For weekly or period measurements (body weight, average daily gain, and blood gas variables) the fixed model effects were diet and period, and random effects were replication and lamb. Data were tested for homogeneity using Bartletts test in Genstat.

## 3. Results

### 3.1. In Vitro Gas Production Study

**Figure 2:**
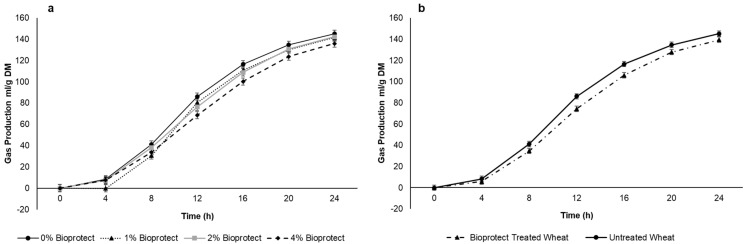
Effect of (**a**) Bioprotect treatment in general (**b**) effect of different dosages of Bioprotect on the cumulative gas production. Gas production curves are representative of 24 h incubation at 39 °C in buffered rumen fluid. The standard error of the difference for the interaction between period and dietary treatments and time is displayed on the on the graph.

After a short lag phase, in vitro gas production increased rapidly before approaching a plateau of approximately 130 mL/g DM after 24 h (Figure 2a,b). There was a linear decrease in the average gas production with the increasing dose of Bioprotect (76.1 vs. 70.5, 72.1 and 67.0 mL/g DM for 0, 10, 20, and 40 g/kg), although there was a Time × Dose interaction (*p* < 0.001) such that the response was greatest between 8 and 16 h (Figure 2a,b). When the data were pooled across the three Bioprotect doses there was a decrease in average gas production (75.9 vs. 69.8 mL/g DM *p* = 0.034) but no difference between doses (*p* = 0.38). Given that there was no difference between doses of Bioprotect, the intermediate dose (20 g/kg) was chosen for the in vivo studies. After 24 h of incubation, the rumen fluid pH did not differ between doses of Bioprotect (*p* = 0.398), but there was a significant difference between 0 h and 24 h of incubation (*p* ≤ 0.001; SED 0.058).

### 3.2. Average Daily Gain, Water, and Feed Intake

As designed, there was no effect of diet on the average daily feed intake (ADFI) (1.19, 1.19, and 1.26 (SED 0.026) kg/d for WD, BD, and MD, *p* = 0.29) (Table 2). In addition, as designed, there was no difference in ADFI between P1 and P2, whereas ADFI was higher during P3 (1.19, 1.14, and 1.32 (SED 0.026) kg/d for P1, P2, and P3, *p* < 0.001). There was no effect of diet on average daily gain (ADG) (15.5 vs. 11.6 and 51.1 (SED 38.4) g/d for WD, BD, and MD, *p* = 0.29), while ADG during P3 tended to be higher than P2 but not different between the other periods (23.2, −6.43, and 80.4 (SED 38.4) g/d for P1, P2, and P3, *p* = 0.083).

There was no effect of diet on water disappearance (4.81, 4.53, and 4.45 (SED 0.182) L/d for WD, BD, and MD, *p* = 0.29) while water disappearance was increased by HS during P2 and P3 (3.57, 4.95, and 5.27 (SED 0.182) L/d for P1, P2, and P3, *p* < 0.001).

### 3.3. Thermoregulatory Responses

**Figure 3:**
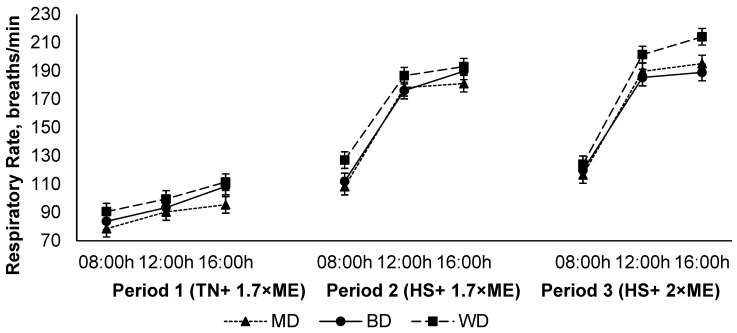
Relationship between respiratory rate, time of observation, and experimental period in sheep fed on different diets. The standard error of the difference for the interaction between period and dietary treatments and time is displayed on the on the graph. The *p*-values for the effects of diet, period, time, diet * period, diet * time, period * time, and diet * period * time were *p* < 0.001, *p* < 0.001, *p* < 0.001, *p* = 0.14, *p* = 0.55, *p* < 0.001, and *p* = 0.17.

The respiration rate increased in response to HS (P1 vs. P2), and was further elevated in P3 (94.6, 161, and 171 (SED 1.8) breaths/min for P1, P2, and P3, respectively; *p* < 0.001) (Figure 3). The respiration rate increased during the day, achieving a maximum late in the afternoon before reducing overnight (108, 156, and 164 (SED 1.8) breaths/min at 09:00, 12:00, and 16:00 h, respectively; *p* < 0.001). Lambs fed the WD had a higher RR than those consuming either the BD or MD across all periods (150, 140, and 137 (SED 4.2) breaths/min for lambs fed WD, BD, and MD, respectively; <0.001). There was an interaction between period and time (*p* < 0.001) such that the RR increased to a greater extent during the hours of high temperature (12:00 and 16:00 h) during HS, especially during P3 (Figure 3).

**Figure 4:**
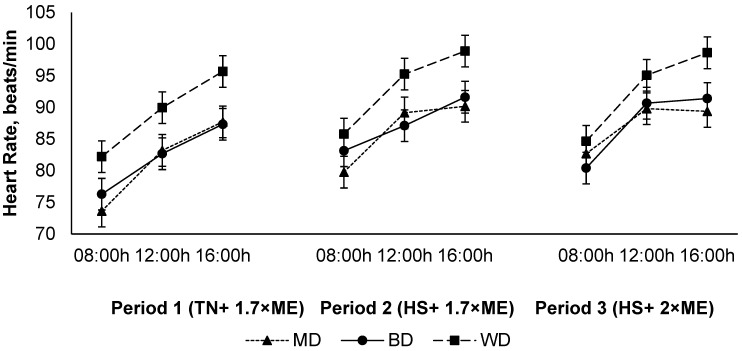
Relationship between heart rate, time of observation, and experimental period in sheep fed on different diets. The standard error of the difference for the interaction between period and dietary treatments and time is displayed on the graph. The *p*-values for the effects of diet, period, time, diet * period, diet * time, period * time, and diet * period * time were *p* ≤ 0.01, *p* < 0.001, *p* < 0.001, *p* = 0.60, *p* = 0.10, *p* = 0.15, and *p* = 0.28.

The heart rate increased in response to HS (P1 vs. P2) with no further increase in P3 (84.3, 89.0, and 89.2 (SED 2.15) beats/min for P1, P2, and P3, respectively; *p* < 0.001) (Figure 4). The heart rate increased during the day, achieving a maximum late in the afternoon before reducing overnight (81.0, 89.2, and 92.3 (SED 0.62) beats/min at 09:00, 12:00, and 16:00 h, respectively; *p* < 0.001). Lambs fed the WD had a higher HR than those consuming either the BD or MD across all periods (91.8, 85.6, and 85.0 (SED 2.15) beats/min for lambs fed WD, BD, and MD, respectively; *p* = 0.011) (Figure 4).

**Figure 5:**
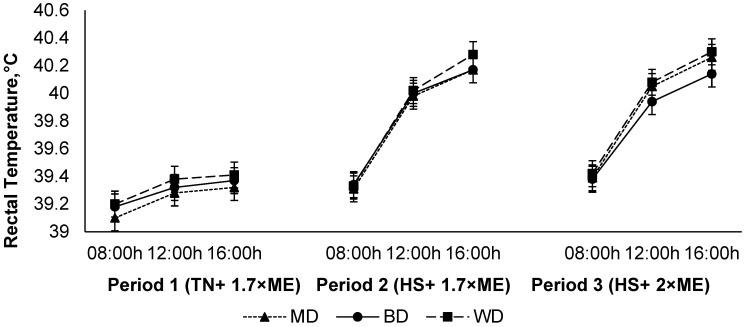
Relationship between rectal temperature, time of observation, and experimental period in sheep fed on different diets. The standard error of the difference for the interaction between period and dietary treatments and time is displayed on the graph. The *p*-values for the effects of diet, period, time, diet * period, diet * time, period * time, and diet * period * time were and *p* = 0.74, *p* < 0.001, *p* < 0.001, *p* < 0.05, *p* = 0.39, *p* < 0.001, and *p* = 0.96.

The rectal temperature increased in response to HS (P1 vs. P2), with a further small increase in P3 (39.28, 39.84, and 39.88 (SED 0.0186) °C for P1, P2, and P3, respectively; *p* < 0.001) (Figure 5). The rectal temperature increased during the day, achieving a maximum late in the afternoon before reducing overnight (39.29, 39.78, and 39.93 (SED 0.0186×) °C at 09:00, 12:00, and 16:00 h, respectively; *p* < 0.001). While there was no main effect of diet on RT (39.71, 39.65, and 39.65 (SED 0.102) °C for lambs fed WD, BD, and MD, respectively; *p* = 0.75), there was a diet × period interaction (*p* = 0.040) such that RT increased to a lesser extent during HS in lambs consuming BD than those consuming the other diets, particularly during the afternoon as evidenced by an interaction between period and time (*p* < 0.001) (Figure 5).

**Figure 6:**
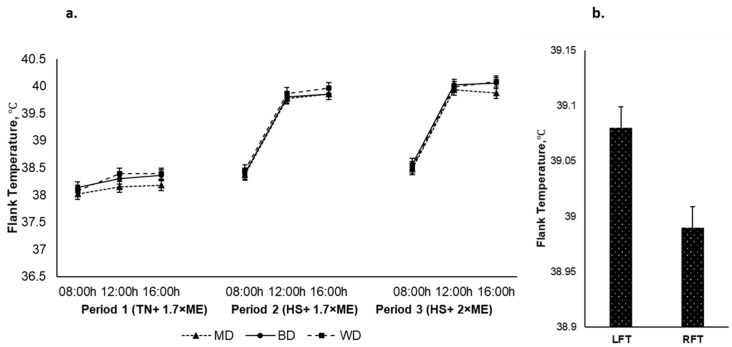
(**a**). Relationship between flank temperature, time of observation, and experimental period in sheep fed on different diets. The standard error of the difference for the interaction between period and dietary treatments and time is displayed on graphs 6. (**b**). Left and right flank temperatures during the experiment. The standard error of the difference for the interaction between period and dietary treatments and time is displayed on the graph. The *p*-values for the effects of diet, period, time, diet * period, diet * time, period * time, and diet * period * time were and *p* = 0.398, *p* < 0.001, *p* < 0.001, *p* = 0.118, *p* = 0. 360, *p* < 0.001, and *p* = 0.857.

The left and right flank temperatures responded qualitatively similarly, although the LFT was higher than the RFT (39.08 and 38.99 (SED 0.019) °C for the LFT and the RFT, respectively, *p* < 0.001). The average flank temperature increased in response to HS (P1 vs. P2) and was further elevated in P3 (38.22, 39.37, and 39.51 (SED 0.023) °C for P1, P2, and P3, respectively; *p* < 0.001) (Figure 6a). The flank temperature increased during the day, achieving a maximum late in the afternoon before reducing overnight (38.33, 39.36, and 39.41 (SED 0.023) °C at 0900, 1200, and 1600 h, respectively; *p* < 0.001). There was an interaction between period and time (*p* < 0.001) such that the flank temperature increased to a greater extent during the hours of high temperature (12:00 and 16:00 h) during HS during P2 and P3. There was no effect of diet on flank temperature nor were there any other interactive effects. However, irrespective of period and dietary treatments the LFT had an upper hand over the RFT Figure 6b.

### 3.4. Blood Biochemistry

While there were no main effects of period or diet on blood pH there was an interaction (*p* = 0.049) such that blood pH increased during P2 and declined during P3 in lambs consuming the WD and the MD but not in those consuming the BD (Table 3). Blood parameters such as pCO_2_, CHCO_3_^−^, cTCO_2_, Na^+^, base excess of blood, and extracellular fluid declined during HS (P1 vs. P2) with a further decline during P3 (*p* < 0.001) but was not affected by diet. The blood K^+^ concentrations did not change across the periods but were lower in lambs fed the WD than the MD (4.22, 4.39, and 4.53 (SED 0.090) mmol/L for the WD, BD, and MD, respectively, *p* = 0.009). The blood anion gap, pO_2_, O_2_ saturation and hematocrit and hemoglobin, glucose, lactate, and Ca^2+^ concentrations were not significantly impacted by either period or diet nor were there any interactions.

## 4. Discussion

This study showed that HS increased thermoregulatory efforts by the lambs, as evidenced by the increased RR and HR. Importantly, the increases in these parameters were reduced through replacing the wheat with Bioprotect-treated wheat or untreated maize. Therefore, reducing the ruminal fermentation rates of wheat starch through Bioprotect treatment can reduce the thermoregulatory responses to HS and provide an alternative to maize concentrates in lamb diets.

The results of this experiment were consistent with basic characteristics of Bioprotect, with reductions in in vitro rumen gas production from the wheat treated with Bioprotect compared to wheat grain. This corresponds with previous results from our laboratory (Dunshea, et al. [28]), where lower fermentation rates of gas production from wheat and barley grains treated with Bioprotect than untreated grains, which would increase the amount of grain starch available for post-ruminal digestion by enzymatic action [17]. Relocating starch fermentation from the rumen to the abomasum has the major advantage of reducing metabolic heat production, which helps animals cope with the rise in temperature [16]. However, there was no difference when applying different dosages of Bioprotect gas production, which is consistent with Gonzalez-Rivas [14], indicating that 1, 2, and 4% dosages of Bioprotect provide a similar level of protection from rumen fermentation. Thus, for the in vivo study, the 2% Bioprotect treatment was selected based on the fact it was a low range dose but could be more easily mixed with the ground wheat.

### Animal Experiment

This experiment quantified the effects of Bioprotect and maize on thermoregulatory responses to HS at equal feed intakes (P1 vs. P2) and at voluntary feed intakes (P2 vs. P3). Because of the short duration of the treatment periods, this experiment was not designed to obtain accurate data on the effect of HS on ADG, so therefore it is not surprising there was no significant effect of HS on ADG when lambs were fed at 1.7× MF. Nevertheless, there was an increase in ADG when feed intake was increased to 2.0× MF, even though they were still under HS conditions.

During HS, sheep and other hairy or woolly animals promote evaporative cooling by increasing their RR [29]. BD- and MD-fed animals showed a much lower RR than WD-fed animals at the peak of HS responses in the late afternoon. During the initial HS period (P2), the RR of BD- and MD-fed lambs were −1.71% and −6.22% lesser than wheat-fed animals, respectively. The greatest difference between treatments in RR was observed during P3 at 16:00 h. Interestingly, BD-fed sheep exhibited better control over respiratory rate than the WD-fed sheep (−12% less RR than WD) at this time. This decrease in the RR of BD-fed lambs presumably indicates an increase in starch escaping the rumen and reaching the omasum and abomasum, which decreases the heat produced during ruminal fermentation. This eventually aids in the alleviation of HS by reducing the metabolic increment of heat that also contributes to total heat load and environmental heat. Our results are consistent with the results reported by Shipandeni [19] who reported a decrease in the in vitro ruminal starch degradability of maize and sorghum grains when treated with Bioprotect. Gonzalez-Rivas, et al. [15] also observed similar trends of greater RR in wheat grain-fed wethers than maize-fed wethers. Further, during the second period of HS, all the lambs increased their RR, and the rate of increase was high during late in the afternoon. In general, the increase in the RR during P3 over P1 and P2 confirms the coupled negative impact of high temperature and increased feed intake during this period.

Heat stress results in a re-distribution of blood flow to the periphery (e.g., the skin) and lungs to aid thermoregulation; the latter can result in a rise in RR [30]. Values for the HR in the present study showed a diurnal rhythmic pattern reaching its maximum during the late afternoon and its minimum during the morning. Our results are in agreement with the previous work from our lab by DiGiacomo, et al. [31] and confirm that the increased HR during severe HS could be part of the general stress response by sheep. An increase in the HR of WD-fed lambs over the BD and MD again strengthens the observations that lambs fed slowly fermentable grains have a better control over metabolic heat production and heat increment than highly fermentable grains such as wheat.

There was no difference in the RT among the different dietary treatment groups. However, throughout this experiment, we documented a daily increase in the RT of lambs from 08:00 h to 120:00 h as the temperature increased in the climatic chambers, with peak RT values reached at 16:00 h. This is attributed to an increased metabolic heat production associated with the time of feeding [32] and environmental heat contributing to accumulating heat load compromising the animal’s ability to maintain core body temperature. These results are consistent with those of Bianca [33] and our previous sheep study (Chauhan et al., 2014) where we consistently reported an increase in the RT from the morning hours to a high RT during the late afternoon and declining in the evening when the temperature was decreased. Interestingly, in this experiment, when animals progressed from P1 to P2, the BD and MD sheep exhibited a smaller increase in RT compared to WD-fed animals. Furthermore, during P3, both MD and WD groups exhibited a greater increase in their RT (0.08 °C and 0.05 °C higher than in P2, respectively). However, the BD group showed a lower RT as compared to both MD and WD groups in P3. Several other studies conducted in different species of small ruminants [5,34] also reported similar results of high RT during HS conditions and is the result of heat produced in body exceeding the heat dissipation from the body and the animal’s inability to maintain thermal balances through various thermoregulatory responses [35]. Skin temperature increases during HS due to thermoregulatory efforts to dissipate heat by radiant heat loss, whereby blood is redistributed to the periphery, where it may dissipate to the environment [35,36]. In the current experiment, increased flank temperatures (both LFT and RFT) were measured after transitioning from thermoneutral (P1) to HS conditions (P2 and P3). Similar to the RT, there were differences in the FT between different time points, such that sheep exhibited higher flank temperatures during 12:00 and 16:00 h of the day than 08:00 h. This is in agreement with Gonzalez-Rivas, et al. [15] who reported that the magnitude of HS determines the increases in the FT during the afternoon while changes in the evening temperature could be attributed as diurnal variations. The left side of the flank is a good indicator of the ruminal temperature, whereas the right side of the flank is a good indicator of the general body temperature [37]. The difference between the LFT and RFT better indicates the heat of fermentation inside the rumen [38]. In the present experiment, we observed a significant increase in the LFT over the RFT, which was significant during the HS (P2 and P3) period.

Heat stress caused a significant change in the blood gas variables, with more prominent effects during P3. However, dietary treatments were not effective in ameliorating changes in blood gas responses, despite their significant influence on the RR and HR. The lower BE(b), and BE(ecf), values have been reported to be as a result of the increased rate of panting under the HS environment or could be due to the decrease in the buffer base in the body fluid of the sheep [39]. During periods two and three compared with P1, there was a fall in the pCO_2_ and ctCO_2_. These changes indicate that the animals experienced hypocapnia during the HS conditions [40]. Irrespective of dietary treatments, the concentration of CHCO_3_^−^ decreased in all lambs with the incidence of HS, which is consistent with the previous findings where they have found a similar decrease in CHCO_3_^−^ concentration with HS exposure. Under the normal comfortable environmental conditions, the bicarbonate buffering mechanism of the animal tries to maintain a ratio of HCO_3_^−^ and pCO_2_ as 20:1 [41]. Our sheep were able to maintain blood pH by adjusting their carbonate and bicarbonate buffering mechanisms [14,42].

## 5. Conclusions

This experiment identified Bioprotect-treated wheat as a suitable feeding strategy to ameliorate HS in lambs. Reducing rumen starch fermentation of wheat through Bioprotect treatment is a feasible alternative to reduce heat increment and resultant heat load, thereby reducing the HS. The HS ameliorative potential of Bioprotect-treated wheat is comparable to that of maize, hence the Bioprotect treatment of wheat grain for animal feeding could be an option for the countries where maize is not a major crop.

## Figures and Tables

**Table 1:** Ingredient and chemical composition of experimental diets offered to Lambs on a DM basis.

	Experimental Diet		
Item	WD	BD	MD
***Ingredient, % DM basis***			
Crushed wheat grain	50		
Bioprotect treated Crushed wheat grain		50	
Crushed maize grain			50
Oaten chaff	25	25	25
Lucerne Chaff	25	25	25
***Chemical composition, DM basis***			
Moisture	10.98	10.98	11.73
Estimated ME, Mcal/kg DM	11.5	11.6	11.7
CP	14.7	14.1	13.1
ADF	16.25	16.25	16.25
NDF	29	29	28.5

**Table 2:** Feed intake, average daily gain, and water disappearance of lambs fed on wheat, Bioprotect treated wheat, and maize-based diets under different periods.

Variables	Diet									*p* Value			
	Wheat (WD)			Bioprotect (BD)				Maize (MD)					
	Period									SED ^1^	Diet	Period	Period * Diet
	P1	P2	P3	P1	P2	P3	P1	P2	P3				
Feed intake, kg/d	1.17	1.12	1.29	1.17	1.11	1.30	1.22	1.19	1.37	0.045	0.29	<0.001	0.84
ADG, g	5.89	−13.0	54.5	31.3	−68.8	72.3	33.0	6.25	114	0.067	0.29	0.29	0.29
Water disappearance, L/d	3.49	5.16	5.79	3.51	4.87	5.20	3.71	4.82	4.82	0.314	0.61	<0.001	0.12

^1^ Standard error of the difference for Diet * Period.

**Table 3:** Comparative assessment of blood gas profile of lambs fed wheat-based diet, Bioprotect treated wheat-based diet and maize-based diet subjected to different experimental periods.

Variables	Diet									Significance			
	Wheat (WD)			Bioprotect (BD)			Maize (MD)						
	Period									SED ^1^	Diet	Period	Period * Diet
	P1	P2	P3	P1	P2	P3	P1	P2	P3				
pCO_2_, mmHg	37.5	34.3	34.3	40.6	36.8	33.2	40.3	35.5	36.0	1.85	0.31	<0.001	0.36
pO_2_, mmHg	35.9	38.3	41.9	36.2	31.8	46.9	34.1	37.2	36.7	6.51	0.83	0.12	0.45
CHCO_3_^−^, mmol/L	27.3	25.7	24.4	28.2	26.0	25.7	28.8	26.7	26.0	0.91	0.21	<0.001	0.91
O_2_ saturation, %	72.1	70.6	75.3	68.1	64.9	73.8	68.5	75.0	72.0	5.52	0.69	0.25	0.40
ctCO_2_, mmol/L	28.4	26.8	25.5	29.4	27.1	26.7	30.0	27.8	27.1	0.94	0.19	<0.001	0.94
Blood pH	7.47	7.49	7.46	7.45	7.46	7.50	7.46	7.48	7.47	0.02	0.99	0.26	0.050
BE(b), mmol/L	3.41	2.25	0.69	3.93	2.01	2.49	4.60	3.75	2.20	1.03	0.29	<0.001	0.40
BE(ecf), mmol/L	3.65	2.31	0.60	4.24	2.13	2.53	5.04	3.25	2.28	1.05	0.34	<0.001	0.50
Anion gap, mmol/L	14.8	14.9	14.8	14.4	14.8	14.4	13.8	14.3	14.4	0.69	0.37	0.67	0.95
Hct, %	23.3	23.4	23.3	24.3	24.4	23.9	24.5	24.1	23.9	1.34	0.76	0.74	0.98
Haem, g/dL	7.93	8.00	7.89	8.21	8.25	8.10	8.36	8.20	8.09	0.44	0.79	0.58	0.96
Glucose, mmol/L	4.13	4.06	4.01	3.86	3.89	3.75	3.84	3.83	3.86	0.20	0.24	0.78	0.95
Lactate, mmol/L	0.75	1.07	0.83	0.96	0.98	0.67	0.76	0.64	0.58	0.31	0.58	0.41	0.75
Ca^2+^, mmol/L	1.30	1.28	1.28	1.28	1.27	1.26	1.29	1.28	1.30	0.02	0.51	0.25	0.76
K^+^, mmol/L	4.18	4.13	4.35	4.40	4.30	4.46	4.68	4.54	4.38	0.15	0.008	0.50	0.19
Na^+^, mmol/L	144.5	142.5	142.4	145.1	143.6	143.4	144.6	143.0	143.4	0.94	0.40	0.001	0.96

^1^ SED: standard error of the difference for Diet * Period.

## Data Availability

Not applicable.
